# Development of a medical academic degree system in China

**DOI:** 10.3402/meo.v19.23141

**Published:** 2014-01-15

**Authors:** Lijuan Wu, Youxin Wang, Xiaoxia Peng, Manshu Song, Xiuhua Guo, Hugh Nelson, Wei Wang

**Affiliations:** 1 Beijing Municipal Key Laboratory of Clinical Epidemiology, School of Public Health, Capital Medical University, Beijing, China; 2 Shekou International SOS Clinic, Shenzhen, China; 3 School of Medical Sciences, Edith Cowan University, Joondalup, Western Australia, Australia

**Keywords:** medical degree, medical education, medical licensing, Bologna Accords, China

## Abstract

**Context:**

The Chinese government launched a comprehensive healthcare reform to tackle challenges to health equities. Medical education will become the key for successful healthcare reform.

**Purpose:**

We describe the current status of the Chinese medical degree system and its evolution over the last 80 years.

**Content:**

Progress has been uneven, historically punctuated most dramatically by the Cultural Revolution. There is a great regional disparity. Doctors with limited tertiary education may be licensed to practice, whereas medical graduates with advanced doctorates may have limited clinical skills. There are undefined relationships between competing tertiary training streams, the academic professional degree, and the clinical residency training programme (RTP). The perceived quality of training in both streams varies widely across China. As the degrees of master or doctor of academic medicine is seen as instrumental in career advancement, including employability in urban hospitals, attainment of this degree is sought after, yet is often unrelated to a role in health care, or is seen as superior to clinical experience. Meanwhile, the practical experience gained in some prestigious academic institutions is deprecated by the RTP and must be repeated before accreditation for clinical practice. This complexity is confusing both for students seeking the most appropriate training, and also for clinics, hospitals and universities seeking to recruit the most appropriate applicants.

**Conclusion:**

The future education reforms might include: 1) a domestic system of ‘credits’ that gives weight to quality clinical experience vs. academic publications in career advancement, enhanced harmonisation between the competing streams of the professional degree and the RTP, and promotion of mobility of staff between areas of excellence and areas of need; 2) International – a mutual professional and academic recognition between China and other countries by reference to the Bologna Accord, setting up a system of easily comparable and well-understood medical degrees.

## Introduction

China has great disparities in personal wealth. The GINI index of wealth maldistribution has reached a figure of 0.47, well above the recognised warning level of 0.4 (1). China's leaders acknowledge the threat to stability that this represents and are promoting a ‘harmonious society’ where people accept being ‘moderately prosperous’ rather than Deng Xiao Ping's ‘to be rich is glorious’ as personal goals. Meanwhile, China's health equities exist between population groups. There is a clear gradient in life expectancy, which increases with prosperity (2). In 2009, the Chinese government launched a comprehensive healthcare reform to tackle challenges to health equities (3).

Medical education will become the key for the successful reform of health (4). Since 1998, the government has made a great effort to expand medical education (5). Currently, there are 159 institutions of higher education for medicine in China (4). The number of students enrolled annually has increased dramatically, rising from 65,695 in 1995 to 149,928 in 2000, and 386,905 in 2005 (6). Meanwhile, China is in the process of transforming its health sector from a situation in which less than 10% of doctors in China were graduates of college level medical education in the early 1970s (7), to a situation in which 64.8% of doctors were graduates of university-level medical education in 2005 (8). Owing to the increased number of graduates with university-level medical education, and increasing social demand for better trained physicians, yearly enrolment into postgraduate medical education increased 6.5-fold from 7,280 in 1998 to 47,412 in 2008 (9).

With the rapidly expanding scale, disparities in the quality of medical education among medical schools have widened substantially. The student to faculty ratio varies from 2:1 to 9:1 in different schools (10). Disparities in the quality of medical education might be helped by increased mobility of both medical students and medical educators, and yet visitors new to China are often struck by the lack of portability of professionals compared to other countries. Although features unique to China, such as resident certificate (*HuKou*) and the old human resource affiliation system (*DanWei*), may contribute to this, it would currently be very difficult for a student to transfer to another medical school mid-studies, or for a graduate to get a job in a hospital they had not trained at – because of a lack of nationwide clear standards, curriculum way-points, and recognised credits for quality of clinical experience.

The situation is similar to how it used to be for western children whose parents’ work required relocation to other cities or countries, and who suffered great disadvantage because of lack of recognition of prior learning and incongruence in primary, middle or high school curricula. This changed with the development of the International Baccalaureate (IB) in 1968 in Geneva. At first it served only to provide a standard for university entry of a few high school students from rich and mobile families. Now it serves nearly 1 million students in 140 countries, and more than half of these students are attending state public schools rather than private elite schools. The benefits are consistent quality and coordinated curriculum and effortless personal mobility. China needs an IB-style reform to allow increased mobility both for students, graduates, teachers and researchers to overcome some of the geographic inequities in medical education. This may be available already in the form of the Bologna Process, which is being adopted in Europe, and other countries including Russia, whose academic and medical traditions have influenced China's past policies.

China's medical degree system appears complex to newcomers from other countries. There are multiple tiers of health care providers, varied routes to accreditation, and diverse roles of personnel with medical degrees in China. These partly reflect the tendency of academics the world over to build and promote their own ‘silos’, but also the difficulties of trying to meet peoples health needs in the face of the major political, economic and social change over the past 80 years. The ‘opening up’ of China's interactions with the outside world, particularly through international trade and communications, has generated a growing awareness of the importance of medical education and cooperation in this field between China and developed countries. This article gives a general overview of the evolution and present state of the medical academic degree system in China. On a global level, mutual understanding of other country's medical academic degree systems is invaluable to future cooperation.

## Evolution of various medical academic degrees in China

Chinese medical education has undergone three distinct periods of development in recent history – before, during and after the Culture Revolution (1966–1976). These changes were driven largely by political rather than educational forces (11).

Before the establishment of the People's Republic of China in 1949, the medical degree system was based on a western European model, introduced by the Republic of China's 1935 ‘Degree Conferral Law’ (12). Academic degrees of medicine were divided into three different levels – bachelors, masters and doctoral. Meanwhile, an 8-year programme leading to the degree of Doctor of Medicine (DM) existed in Peking Union Medical College (PUMC) established in 1917 with the support of the Rockefeller Foundation. Since the establishment of the People's Republic of China in 1949, the evolution of the medical degree system has been somewhat turbulent and uneven. The three-level system was disbanded in favour of a pre-cultural revolution system (1949–1965). Medical education was delivered by secondary medical schools (SMS) and medical universities. The SMS enrolled graduates of junior high (9 years) education for a period of study lasting 3–4 years. The medical universities enrolled graduates of high school education (12 years) for a period of study of 5–6 years (13). Medical universities began to enrol postgraduates from university-level medical education for a period of study of 4 years, equal to the *Kandidate nauk* degree of the Soviet Union. None of these programmes awarded formal degrees.

With the onset of the Cultural Revolution (1966–1976), medical education essentially ceased. Peer-chosen ‘worker–peasant–soldier’ students were enrolled to learn a curriculum emphasising political ideology and practical training over medical science. At this stage, entrance examinations, as well as degrees, were abolished.

After the Cultural Revolution, a modern medical degree system came into effect in 1981. A three-level medical degree system – Bachelor of Medicine (BM), Master of Medicine (MM), and Doctor of Medicine (DM) – was adopted (14). In 1988, a 7-year programme was adopted to give medical students the opportunity to pursue other fields alongside their medical studies with the aim of broadening their knowledge and appreciation of the humanities and enhancing their career skills. Successful completion of the 7-year course led to the award of the MM. As mastery of clinical skills is essential in the professional development of doctors who care for patients, it was decided in 1997 to separate and recognise two types of medical academic degree at both master and doctoral levels, i.e., a clinical professional degree and a research degree (15). This meant that the medical degree system became more complex. To train medical scientists and medical educators for China, an 8-year programme leading to the degree of DM was initiated in 2001 (16). The first class of 293 graduates of an 8-year programme obtained this degree in 2009. This degree has existed in PUMC since 1917, although its output was modest (only 313 graduates between 1925 and 1949). Nevertheless, its graduates have had a profound impact on the establishment of western-style medicine and scientific research in China (17, 18).

## Present state of medical academic degree system in China

Unlike medical degrees in the UK, e.g., Bachelor of Medicine and Bachelor of Surgery (MBChB), which can only be awarded to medical students, medical degrees in China can also be awarded to students specialising in other fields, e.g., Human Movement Science, History of Science and Technology, Biomedical Engineering and Social Medicine and Health management. This means that the medical degree system in China is more complex than in most countries (19). For the sake of clarity in understanding the nature of a medical degree in China, this article will focus on one specialty (clinical medicine).

Firstly, an overview of the levels, the route and the form of medical degrees will be examined. Secondly, the relationship between the clinical training provided in the professional degree stream and the residency training programmes (RTPs) stream will be analysed. Thirdly, as in most other countries, a license to practise medicine is a separate issue from attainment of a medical degree. This section will focus both on medical degrees and medical licensing. Finally, the role of these qualifications in a doctor's career will be considered.

## Medical degrees: multilevel, multiroute and multiform approach

**Fig. 1:**
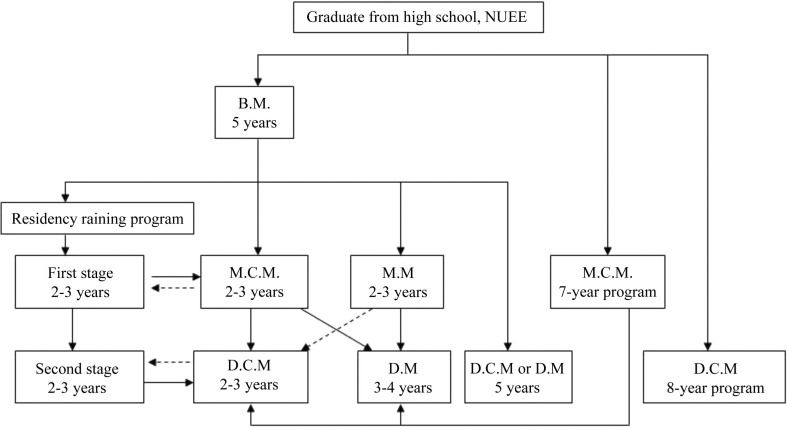
Multipath to acquire multilevel of medical degrees.

In the past decade, China has struggled to balance the need of its medical educational strategy, as shown by the scale (6, 9) and complexity of the medical academic degree system (Fig. 1).

### The clinical professional degree stream

The medical academic degree system in China is organised around a three-level degree system awarding bachelor, master and doctorate degrees. Medical universities provide a 5-year undergraduate medical curriculum for candidates who have completed high school education and passed the National Admission Examination, leading to the BM.

Holders of the BM who pass the National Postgraduate Entrance Examination are qualified to enter the master's degree programme (professional degree or research degree) for 2–3 years of full-time study.

After obtaining the master's degree, a student who passes the National Doctor Entrance Examination may enter a medical university or medical institute to pursue a doctorate degree. The DM is a research degree. It is awarded after successful completion of 3 years of further study: 6-month curriculum study and 2.5-year medical research. Also, the Doctor of Clinical Medicine (DCM) is a professional degree. It is awarded after successful completion of 3-year study, 6-month curriculum study and 1.5-year clinical training in a hospital and 1-year medical research. The DCM highlights training in clinical skills. It is roughly equal to the Doctor of Medicine (MD) in the USA, or a Fellowship in a Specialist College in UK or Australia. The graduates are required to have solid knowledge in the diagnosis and treatment of special diseases and have specific skills in a subspecialty, taking on the responsibility as chief-of-residents, supervising and training junior clinical staff. They should also do clinical research, write a dissertation, and be proficient in reading English. They will become physicians. The study period between graduating from high school and obtaining DCM varies from eight to 14 years in China.

In addition to the 5-year undergraduate medical curriculum, some medical universities present 7-year programmes in clinical medicine leading directly to a master's level qualification, i.e., Master of Clinical Medicine (MCM). More than 40 medical schools have both 5- and 7-year undergraduate medical courses in China (20).

The top 12 leading medical schools in China have been authorised by the Ministry of Education to develop a dedicated 8-year programme, leading to the DCM. Yearly enrolment into the 8-year programme in the 12 medical schools is limited to 1,300 individuals (16).

### The research degree

A professional degree differs from the research degree in the area of professional supervision and discipline. Table 1 shows the difference between these two types of medical degree. The MM and DM are all research degrees. Medical graduates holding research degrees will perform research in the fields of Medicine as their primary professional activity. Publications in peer-reviewed national journals are considered essential requirements for master graduates, while publications in peer-reviewed international journals are considered essential requirements for doctorate graduates. The DM is roughly equivalent to the PhD qualification in the USA or UK. They will become physician–scientists who perform medical research as their primary professional activity.

**Table 1:** Difference between professional degrees and research degrees in medical sciences

	Professional degree	Research degree
Field of study	Professional discipline	Academic discipline
Levels of medical degree awarded	Master's level; doctorate level	Master's level; doctorate level
Outputs	Senior doctors, senior public health physicians, senior dentists, senior pharmacist, senior nurses	Physician–scientists who perform medical research as their primary professional activity
Titles for medical degree	Clinical Medicine (MCM/DCM)	Pre-clinical Medicine
	Public Health (MPH)	Clinical Medicine
	Stomatology (MSM/DSM)	Preventive Medicine
	Pharmacological Science (MPS/DPS	Dentistry
	Chinese Materia Medica (MCMM/DCMM)	Pharmacological Science
		Human Movement Science
		History of Science and Technology
		Biomedical Engineering
		Social Medicine and Health Management (all awarded MM/DM)

MCM = Master of Clinical Medicine; MCMM = Master of Chinese Materia Medica; MPH = Master of Public Health; MPS = Mater of Pharmacological Science; MSM = Master of Stomatological Medicine; DCM = Doctor of Clinical Medicine; DCMM = Doctor of Chinese Materia Medica; DPS = Doctor of Pharmacological Science; DSM = Doctor of Stomatological Medicine.

## The relationship between the professional degree and residency training programme streams

It was recognised that there was a need for an independent body to accredit the clinical training of other bodies across China because many graduates of the professional degree stream had inadequate clinical experience. This was due to the great disparity in the quality of medical education across China. To address this, the RTP was initiated in 1993, under the responsibility and supervision of the Ministry of Health. In 1995, the Ministry of Health established the Council for Graduate Medical Education to set the standards for residency training and give accreditation to residency training centres. There are 2400 such centres in 26 of China's 34 provincial level administrative areas. Maldistribution of equipment and resources means inevitable disparity in the quality of clinical training available.

The RTP is organised into two stages: stage-I includes 3 years to work on ‘medicine or surgery in general’ with accreditation before starting stage II. The trainees are expected to reach the level of Specialist Physician or Surgeon. Stage II lasts between 2 and 3 years. Within this training, the trainees are expected to become competent to independently consult and treat routine and emergency cases.

The professional degree structure recognises RTP clinical training as being essential to high-level professional development. The clinical training objective endpoint of the professional degree stream is intended to be consistent with the RTP. The clinical skills of graduates with MCM are expected to achieve stage-I of RTP. After completion of stage-I of RTP, the physician may apply for MCM, which includes a 1-year master's curriculum, proficiency in a foreign language (usually English) and a comprehensive examination for the master's degree organised by the Ministry of Health, then defending a dissertation in the fourth year. After completion of stage II of RTP the physician may apply for DCM, which includes completing a doctor's curriculum in 1 year, passing the foreign language examination for a doctor's degree, and defending a dissertation.

Although the professional degree has prestige, in fact many of its graduates must repeat training years to gain RTP accreditation. Obtaining RTP level one or two in Beijing requires a combination of a medical degree as well as clinical working experience (21). It is pertinent to note that trainees with MCM without any working experience need to complete 2 years of the RTP stage-I before they may progress to stage II. Trainees with 2–5 years’ working experience need to complete 1 year of RTP stage-I before progression to RTP stage II.

The lack of mutual recognition of training in the professional degree stream and the RTP stream leads to repetition and wasteful duplication of clinical training resources. The framework regulating the relationship between the professional degree and the RTP is a matter of national debate. Work has been done in Peking University Health Science Centre (PUHSC) to standardise and unify the clinical training as well as the assessment of clinical skills for trainees in both Professional Degree and RTP streams. Thus, the PUHSC graduate holding MCM is allowed to proceed directly to RTP stage II (22).

## Other medical qualifications

A first degree is a prerequisite for entry to many professions, but a BM is not essential to apply for a medical license in China. In addition to the degree-oriented medical education, there are also two kinds of medical programmes: The first is a high school level, 3-year vocational training which is now being phased out. The second is a university-level, 3-year programme leading to a certificate.

In order to obtain a license to practice medicine, graduates must pass the National Medical Licensing Examination (NMLE) (23) and have hospital experience. The BM graduates must have 1 year of hospital experience, certificate graduates have 2 years, and vocational training programme graduates must have 5 years of hospital experience.

## The role of medical qualifications in doctor's careers

With an excess in the production of health workers over the absorption into the health workforce (4), it is difficult to find a job for a medical graduate. Higher degrees are instrumental in career advancement, including employability in urban hospitals – a potent motivator for would-be doctors to obtain degree-oriented medical education.

China has developed its own nomenclature for physicians, i.e., junior doctor, doctor in charge, assistant chief doctor and chief doctor. It is a stepwise system in which graduates holding the BM progress from junior doctor to chief doctor within a period of 15 years. Although degree qualifications are not necessary to obtain a license for medical practice, higher level degrees are necessary for rapid promotion (24). For example, without the 8 years’ DCM study, it is not possible to be appointed as a chief doctor until the age of 39 years. On the other hand, DCM degree holders might be appointed as young as 33 years (25).

## A need for mutual recognition of degrees has been acknowledged internationally

In May 1998, a conference attended by 2000 academics to mark the 800th anniversary of the founding of Sorbonne University led to a declaration which focused on a progressive harmonisation of framework of degree courses, common levels for bachelors, masters and doctorates (also known as the 3 + 2+3 system), and increased international mobility of students, teachers and researchers, to promote a ‘Europe of Knowledge’ not just of commerce. Other principles have been defined, such as life-long learning, and a system of objective credits to lend weight to vital but non-academic accomplishments like practical engineering or clinical medical experience (26). The number of countries supporting this undertaking by signing the Bologna Declaration grew from 26 in 1999 to 46 (27 European countries and 19 non-European countries) in 2009 (27). Medical education in recent decades has evolved from a strict separation between academic and clinical years, towards patient oriented clinical integration in the whole syllabus. This is creating better doctors, but makes implementing Bologna style transferable modules more challenging. However, seven countries have already enacted legislation to do this in some form (28). The ‘3 + 2+3’ system might fit naturally when a pre-medical bachelor's degree is already required, as in the USA and Canada and increasingly in Australia, UK and Ireland medical schools. By contrast, France and some of Britain's schools accept high school graduates after a year of Foundation study with over 50% attrition, then a monolithic study period towards a medical degree. Despite the challenges, all medical schools want international recognition of the significance and quality of the degrees they issue. There is a European consensus on the matter that harmonisation of medical education in Europe is crucial whatever system exists (29).

The Chinese government has always attached great importance to enhancing the mutual understanding of higher education sectors and advancing the exchanges and cooperation between China and other countries. In 1983, China signed an agreement with 19 other countries to promote regional and worldwide cooperation in the matter of comparability and recognition or equivalence of studies and academic degrees. In addition to this document, China has signed the agreement of mutual recognition of Studies, Diplomas and Degree in Higher Education with 26 countries, e.g., USA since 1998.

## The future

China's medical degree system has undergone significant changes of reform, readjustment and development over the last eight decades. The current multilevel, multiroute and multiform approach to medical degree education has been driven by society's demands and advances in scientific knowledge. Compared with the readily understood medical degree system in the USA/Europe, the diversity of the current degree-oriented medical education, and the multiple routes taken to acquire diverse kinds of medical degree remain potentially confusing for students, employers and indeed for universities in China. Whether or not a three-level degree system continues to be used for medical education, consideration must be given to the need for harmonisation of medical academic degrees, for standards-based recognition of clinical experience of trainees in both the Professional Degree and RTP streams, and the relationship between medical degrees, licensing to practice, and career development.

Some of Bologna's goals and specific agencies might be beneficial to China. Firstly, an agency whose duty is to promote and enable mobility of students and teachers between different parts of China, and getting extra academic credit and extra pay for doing exchanges. Participation should enhance rather than hinder the individual's career.

Secondly, a credit transfer and accumulation scheme similar to the ECTS – to transparently and objectively weight the value of clinical experience for nationwide cooperation in quality assurance. Thirdly, recognised ‘way points’ during the course of study where transfer to another location or institution may occur. These ‘way points’ could fit the ‘3 + 2+3’ pattern. This can only happen when curricula are congruent and accreditation at each level is transparent, objective and recognised across China.

These would have a powerful effect to decrease disparity in the quality of medical education and quality of service delivery across China. China's leaders in education already recognise the need for this kind of process (30).
